# Long-term prognostic value of cardiopulmonary exercise testing in patients with hypertrophic cardiomyopathy

**DOI:** 10.1093/eschf/xvag013

**Published:** 2026-01-22

**Authors:** Leopoldo Ordine, Grazia Canciello, Salvatore Di Napoli, Roberto Polizzi, Daniela Pacella, Raffaella Lombardi, Giovanni Esposito, Maria-Angela Losi

**Affiliations:** Department of Advanced Biomedical Sciences, University of Naples ‘Federico II’, School of Medicine, Via S Pansini, 5, Naples I-80131, Italy; Department of Advanced Biomedical Sciences, University of Naples ‘Federico II’, School of Medicine, Via S Pansini, 5, Naples I-80131, Italy; Department of Advanced Biomedical Sciences, University of Naples ‘Federico II’, School of Medicine, Via S Pansini, 5, Naples I-80131, Italy; Department of Advanced Biomedical Sciences, University of Naples ‘Federico II’, School of Medicine, Via S Pansini, 5, Naples I-80131, Italy; Department of Public Health, University of Naples ‘Federico II’, School of Medicine, Via S Pansini, 5, Naples I-80131, Italy; Department of Advanced Biomedical Sciences, University of Naples ‘Federico II’, School of Medicine, Via S Pansini, 5, Naples I-80131, Italy; Department of Advanced Biomedical Sciences, University of Naples ‘Federico II’, School of Medicine, Via S Pansini, 5, Naples I-80131, Italy; Department of Advanced Biomedical Sciences, University of Naples ‘Federico II’, School of Medicine, Via S Pansini, 5, Naples I-80131, Italy

**Keywords:** Hypertrophic cardiomyopathy, Heart failure, Cardiopulmonary exercise testing, Exercise capacity, Prognosis, Preventive cardiology

## Abstract

**Introduction:**

Hypertrophic cardiomyopathy (HCM) is a heterogeneous myocardial disorder characterized by left ventricular hypertrophy. The role of cardiopulmonary exercise testing (CPET) in predicting major adverse cardiac events (MACE) remains incompletely understood, particularly over long-term follow-up and independently of baseline symptoms.

**Methods:**

We longitudinally studied 154 HCM patients (age 43 ± 16 years; 27% female), who underwent symptom-limited CPET. At baseline, 98 patients were in New York Heart Association (NYHA) Class I, 48 in Class II, and 8 in Class III. Septal reduction therapies (SRT), progression to end-stage HCM (ES-HCM), sudden cardiac death (SCD), heart failure-related death (HF), and heart transplantation (HT) represented a composite MACE endpoint.

**Results:**

Over a mean follow-up of 12 ± 9 years, 38 patients experienced MACE (SRT = 9; ES-HCM = 11; SCD = 10; HF/HT = 8). In multivariable analysis, independent predictors of MACE were percentage predicted peak VO_2_ (*P*VO_2_%) < 60 [hazard ratio (HR) 4.16, 95% confidence interval (CI) 1.89–9.14; *P* < .001], and NYHA Class >I (HR 2.27, 95% CI 1.06–4.89; *P* = .036). By using SRT as a competing risk, the only predictor of MACE became *P*VO_2_% < 60 (HR 3.966, 95% CI 1.626–9.670; *P* = .002). Among asymptomatic patients (i.e. NYHA Class I), only *P*VO_2_% < 60 remained a significant predictor of MACE (HR 5.611, 95% CI 1.635–19.253; *P* = .006), with risk divergence evident after nearly 15 years of follow-up. The result was also confirmed in the competing risk analysis.

**Conclusion:**

In this long follow-up study, CPET is a powerful prognostic tool in HCM. A reduced peak VO_2_ identifies those at higher risk, highlighting the potential for CPET to improve risk stratification, even among patients classified as NYHA Class I.

## Graphical Abstract

**Graphical Abstract:**
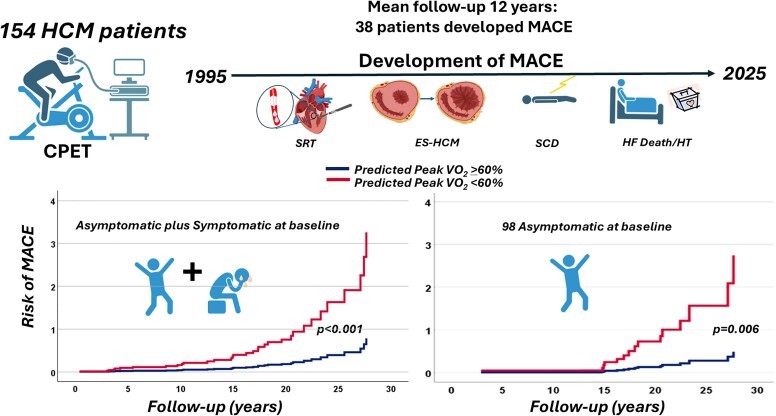
Study protocol and results. A percentage predicted peak VO_2_ of <60%, as assessed by cardiopulmonary exercise testing, is a strong predictor of major adverse cardiovascular events over long-term follow-up in patients with hypertrophic cardiomyopathy, including those who are asymptomatic. CPET, cardiopulmonary exercise testing; ES-HCM, end-stage hypertrophic cardiomyopathy; HCM, hypertrophic cardiomyopathy; HF, heart failure death; HT, heart transplantation; MACE, major adverse cardiac events; SCD, sudden cardiac death; SRT, septal reduction therapy.

## Introduction

Hypertrophic cardiomyopathy (HCM) is the most common inherited cardiovascular disorder, characterized by left ventricular (LV) hypertrophy that cannot be solely attributed to abnormal loading conditions.^1^ The clinical presentation of HCM is complex and varies widely; individuals may exhibit different symptoms and severity, even among family members carrying the same genetic mutation. This variability makes the expression of this condition and its effects difficult to predict.^1^ Contemporary management and therapeutic innovations have changed the clinical course, improving prognosis.^2,3^ Nevertheless, HCM is still marked by higher morbidity and mortality rates compared with the general population, with a significant risk of septal reduction therapy (SRT) sudden cardiac death (SCD), heart failure (HF) death, and the need for heart transplantation (HT).^2,4^ These conditions often emerge later in life, emphasizing the necessity for continued surveillance and a more personalized risk assessment, even for younger and asymptomatic patients.

Cardiopulmonary exercise testing (CPET) is a non-invasive method that thoroughly assesses exercise performance, offering diagnostic and prognostic information for cardiovascular and pulmonary diseases.^5^ By integrating the advantages of an exercise test with the simultaneous measurement of respiratory gases, CPET stands as a crucial tool for evaluating HCM patients, providing valuable information for diagnosis, differential diagnosis, prognosis, and management.^6–10^ International guidelines on HCM recommend conducting CPET in patients with severe symptoms who are being evaluated for HT or mechanical support.^2,11^ However, CPET is not considered a routine part of the evaluation for all HCM patients, especially those who are asymptomatic. Additionally, the role of CPET in predicting major adverse cardiac events (MACE) is not yet fully understood.^2,11,12^ In this study, we present our single-centre experience, focusing on the prognostic role of CPET during long-term follow-up in both symptomatic and asymptomatic HCM patients.

## Methods

### Population

Consecutive patients with HCM referred and followed in the HCM outpatient clinic of the University Hospital Federico II (Naples, Italy) were enrolled. For the present study, we included HCM patients aged ≥18 years, with LV ejection fraction (EF) >50%, capable of performing CPET without any contraindications, without coronary artery disease (CAD) history, and with at least 6 months of follow-up. The diagnosis of HCM was based on echocardiographic identification of LV maximal wall thickness (MWT) ≥15 mm (or ≥13 mm in case of positive family history), unexplained by abnormal loading conditions.^2,13^ Phenocopies such as cardiac amyloidosis or Fabry disease were excluded. The study conforms to the ethical standards of the Declaration of Helsinki, and written informed consent was obtained from all participants.

### General assessment

Each HCM patient was evaluated by a cardiologist with a comprehensive clinical assessment, including history, pedigree, New York Heart Association (NYHA) functional class, 12-lead electrocardiogram (ECG), 48 h ECG Holter monitoring, transthoracic 2D and Doppler echocardiography, genetic testing, and CPET. In the genetic testing, all identified variants, including both pathogenic/likely pathogenic variants and variants of uncertain significance (VUS), were classified using the ClinVar database.^14^ Cardiac magnetic resonance (CMR) has been consistently performed in recent years, whereas in the early years of our clinic, it was less available.

### Echocardiography

As previously reported, a comprehensive colour Doppler echocardiogram was performed.^13,15,16^ Briefly, the following echocardiographic measurements were considered: body surface area (BSA)-indexed left atrial volume (LAVi), mitral *E*/*e*′, MWT defined as the greatest thickness measured at any LV segment, LVEF with Simpson’s biplane methods, maximal LV outflow tract obstruction (LVOTO) at rest and after the Valsalva manoeuvre. Significant LVOTO was defined by a maximal LVOT gradient ≥30 mmHg either at rest or after the Valsalva manoeuvre.

### Cardiopulmonary exercise testing

Symptom-limited CPET was performed in each patient using an electronically braked cycle ergometer (Medical Graphics, St. Paul, MN, USA) according to published guidelines.^5^ All medications administered during the CPET were documented. Medications were not withheld before the test. Height and weight were recorded, and body mass index was calculated at the time of CPET. Before each test, the equipment was calibrated using standard reference gases. All CPETs were performed as previously reported.^17^ All patients exercised at a constant speed of 60 revolutions per minute (rpm) for 3 min. After this initial period, the workload was increased by 1 W every 3 s, following a ramp protocol. This increase continued until patients either reported shortness of breath or fatigue. The ramp exercise protocol was tailored based on each patient's age and reported exercise capacity. A breath-by-breath analysis of expiratory gases was performed, and peak oxygen consumption (Peak $\dot{V}O_{2}$) was defined as the 10 s averaged peak exercise value of oxygen consumption. The percentage predicted peak VO_2_ (*P*VO_2_%) was determined by using the Wasserman/Hansen equations.^6^ The peak respiratory exchange ratio was defined as the ratio between carbon dioxide production $\left(\dot{V}CO_{2}\right)$ and $\dot{V}O_{2}$ at peak exercise. The ventilation/carbon dioxide output $(\dot{V}E/\dot{V}CO_{2})$ slope was obtained by linear regression analysis of the data acquired throughout the entire exercise period. The anaerobic threshold (AT) was determined using the V-slope method by identifying the point at which $\dot{V}CO_{2}$ increased disproportionately relative to $\dot{V}O_{2}$ All patients underwent continuous 12-lead ECG monitoring and noninvasive blood pressure measurements during testing.

### Endpoint

Major adverse cardiac events represented the endpoint. Major adverse cardiac events was a composite of SRT, progression to end-stage (ES)-HCM, SCD, HF death, and HT. Septal reduction therapy, including ventricular septal myectomy and alcohol septal ablation, was indicated according to international guidelines in obstructive HCM patients who were still symptomatic (NYHA III and IV) despite receiving optimal medical therapy.^2,11^ End-stage-HCM was defined by the development and persistence of a reduced LVEF of <50%, along with worsening HF symptoms that necessitate conventional HF medical therapy.^11,18^ Sudden cardiac death was defined as sudden and unexpected death occurring within 1 h of the onset of symptoms, or as death in patients found dead within 24 h of being asymptomatic, with the presumed cause being a cardiac arrhythmia or haemodynamic catastrophe.^19^ Aborted SCD was considered equivalent to SCD and was defined by the appropriate therapy from an implantable cardiac defibrillator, triggered by ventricular fibrillation or rapid ventricular tachycardia at >180 b.p.m.^2,11^ Death related to HF was defined as death occurring after at least 1 h of signs and symptoms of HF or following cardiogenic shock.^2,11^

### Statistical analysis

Results are presented as mean ± standard deviation for continuous variables and as number of cases with corresponding percentages for categorical variables. All statistical analyses were performed using IBM SPSS Statistics version 29 and R statistical software version 4.4.0. The follow-up duration for each patient was determined from the date of their CPET to the date of their most recent visit. Univariate analyses were conducted using the Cox proportional hazards model to examine the association between predictor variables and time-to-event outcomes. Patients were categorized based on the *P*VO_2_% (<60 and ≥60).^9,20–23^ Variables that showed a significant association with the outcome in univariate analysis were selected for inclusion in the multivariate model. As sensitivity analyses, models were computed separately considering *P*VO_2_% as a dichotomous and as a continuous predictor. Moreover, a separate adjusted model was also computed to investigate the potential non-linearity of the association between continuous *P*VO_2_% and the time-to-event outcome using restricted cubic splines with knots at conventional thresholds (20%, 40%, 60%, 80%). In a separate adjusted model, the interaction term between dichotomous *P*VO_2_% < 60 and LVOTO was added to investigate the need for stratified analyses.

Finally, an additional analysis was done, excluding patients who only presented with SRT during follow-up. However, because the main outcomes of interest, ES-HCM, SCD, HF death, and HT, can occur after earlier events like SRT, we run another Cox regression analysis. In this additional model, the SRT that occurred before the main outcomes was treated as a competing risk event. To account for this, we censored any SRT that happened before the primary outcomes when evaluating the effect of the main predictor.^24^ Variables were considered statistically significant if their *P*-values were <.05.

## Results

### Patients’ characteristics

Out of 222 HCM patients who underwent CPET, 15 were excluded because the CPET was performed after SRT, 12 were excluded due to LVEF <50%, 3 were excluded due to a CAD positive history, and 38 were excluded due to a follow-up period shorter than 6 months. Thus, the final population consisted of 154 patients (age 43 ± 16, 27% women). The baseline characteristics of the study population are listed in *Table 1*. Ninety-eight patients (64%) were in NYHA Class I. Sixty-six patients (43%) had significant LVOTO, 44 observed at rest and 22 elicited only after the Valsalva manoeuvre (*Table 1*). Eighty-nine patients underwent genetic testing, which identified pathogenic or likely pathogenic sarcomeric variants in 55 patients (36%). In most of the remaining 34 patients, VUSs were detected (*Table 1*). Forty-eight patients performed CMR, with 40 of them showing late gadolinium enhancement (LGE).

**Table 1:** Baseline characteristics of the study populations

Variable	Overall population (=154)
Age (years)	43 ± 16
Age at diagnosis (years)	36 ± 17
Female sex, *n* (%)	41 (27)
Family history of HCM, *n* (%)	97 (63)
Family history of suddendeath, *n* (%)	51 (33)
Hypertension, *n* (%)	19 (12)
Obesity, *n* (%)	16 (10)
NYHA functional Class >I, *n* (%)	56 (36)
Sarcomeric mutation, *n* (%)	55 (36)
Maximal LV wall thickness (mm)	21 ± 5
LAVi ≥34 ml/m^2^, *n* (%)	46 (34)
*E*/*E*′	9 ± 4
LV ejection fraction (%)	67 ± 5
LVOTO ≥30 mmHg, *n* (%)	66 (43)
LVOTO ≥30 mmHg at rest, *n* (%)	44 (29)
LVOTO ≥30 mmHg after Valsalva manoeuvre, *n* (%)	22 (14)
Peak VO_2_ (ml/kg/min)	23 ± 8
Percentage predicted peak VO_2_ (%)	74 ± 20
Percentage predicted peak VO_2_ > 100%, *n* (%)	17 (11)
Percentage predicted peak VO_2_ < 80%, *n* (%)	104 (67)
Percentage predicted peak VO_2_ < 60%, *n* (%)	38 (25)
VE/VCO_2_ slope	28 ± 5
AT (% peak VO_2_)	17 ± 9
Therapy (BB, CCB, ACEi/ARB, diuretics, antiarrhythmics), *n* (%)	97 (63)
Beta-blockers, *n* (%)	59 (38)
Calcium-channel blocker, *n* (%)	42 (27)
ACEi/ARB, *n* (%)	8 (5)
Diuretic agents, *n* (%)	7 (4)

ACEi, angiotensin-converting enzyme inhibitor; ARB, angiotensin II receptor blocker; AT, anaerobic threshold; HCM, hypertrophic cardiomyopathy; LAVi, indexed left atrial volume; LV, left ventricular; MACE, major adverse cardiac events; NYHA, New York Heart Association; Peak VO_2_, peak oxygen consumption; VE/CO_2_ slope, minute ventilation (VE) to carbon dioxide production (VCO_2_) slope.

### Cardiopulmonary exercise

The cardiopulmonary exercise test was terminated by shortness of breath and/or fatigue in all patients. Eleven patients had exercise-induced hypotension. However, none of the patients interrupted the exercise test prematurely because of a decrease in systolic blood pressure below 100 mmHg. At the time of CPET, 5% of patients were taking angiotensin-converting enzyme inhibitors or angiotensin receptor blockers, while 38% were on beta-blockers and 27% on non-dihydropyridine calcium channel blockers. The mean value of the *P*VO_2_% was 74 ± 20, with significant differences between patients in NYHA Class I and those in NYHA Class ≥II (76 ± 21% vs 70 ± 16%, *P* = .037). Patients with significant LVOTO had a lower *P*VO_2_% than patients without (70 ± 17 vs 77 ± 21, *P* = .015), while there were no differences between sarcomeric and non-sarcomeric patients (73 ± 20 vs 71 ± 18, *P* = .693). Beta-blocker use did not differ significantly between patients with or without low *P*VO_2_% (50% of those with *P*VO_2_% < 60 and 35% of patients with *P*VO_2_% ≥ 60, *P* = .088). In the context of the 48 patients who performed CMR, 40 showed LGE with their *P*VO_2_% similar to those without LGE (75 ± 21% vs 66 ± 22%, *P* = .302). Other cardiopulmonary exercise test variables are reported in *Table 1*.

### Outcome

The mean follow-up period was 12 ± 9 years (interquartile range: 5–19 years), with 55% of patients followed for more than 10 years and 20% for more than 20 years. During follow-up, 38 patients experienced MACE, with specific distributions as follows: 9 had SRT, 11 developed ES-HCM, 10 patients had SCD, 8 had HF death or HT. Baseline patient clinical and instrumental data for those who experienced MACE compared with those who did not are shown in *Table 2*. Patients with the worst outcomes were more symptomatic, demonstrated lower exercise capacity and AT, and had a higher likelihood of carrying a sarcomere mutation. Additionally, these patients more frequently displayed significant LVOTO, as well as greater MWT and LAVi (*Table 2*). There were no significant differences in age, age at diagnosis, sex, family history of HCM and SCD, VE/VCO_2_ slope, or comorbidities such as obesity and hypertension (*Table 2*).

**Table 2:** Baseline differences between patients who developed major adverse cardiac events and those who did not during follow-up

Variable	No MACE (116)	MACE (38)	*P*
Age (years)	42 ± 16	44 ± 15	.258
Age at diagnosis (years)	35 ± 17	36 ± 17	.387
Female sex, *n* (%)	30 (26)	11 (29)	.709
Family history of HCM, *n* (%)	71 (61)	26 (68)	.424
Family history of sudden death, *n* (%)	36 (31)	15 (40)	.337
Hypertension, *n* (%)	14 (12)	5 (14)	.830
Obesity, *n* (%)	13 (11)	3 (8)	.561
NYHA functional Class >I, *n* (%)	36 (31)	20 (53)	.016
Sarcomeric mutation, *n* (%)	36/65 (55)	19/24 (79)	.040
LAVi ≥34 ml/m^2^, *n* (%)	37 (32)	19 (55)	.018
Mitral *E*/*e*′	9 ± 5	7 ± 4	.123
Maximal LV wall thickness (mm)	20 ± 5	23 ± 5	.005
LV ejection fraction (%)	67 ± 5	66 ± 6	.402
LV outflow tract obstruction ≥30 mmHg, *n* (%)	38 (33)	28 (74)	<.001
Peak VO_2_ (ml/kg/min)	24 ± 8	20 ± 6	.004
Percentage predicted peak VO_2_ (%)	77 ± 20	66 ± 17	.004
Percentage predicted peak VO_2_ < 60%, *n* (%)	23 (20)	15 (39)	.015
VE/VCO_2_ slope	29 ± 5	29 ± 6	.465
AT (% peak VO_2_)	18 ± 10	13 ± 4	.004

AT, anaerobic threshold; HCM, hypertrophic cardiomyopathy; LAVi, indexed left atrial volume; LV, left ventricular; MACE, major adverse cardiac events; NYHA, New York Heart Association; Peak VO_2_, peak oxygen consumption; VE/CO_2_ slope, minute ventilation (VE) to carbon dioxide production (VCO_2_) slope.

**Figure 1:**
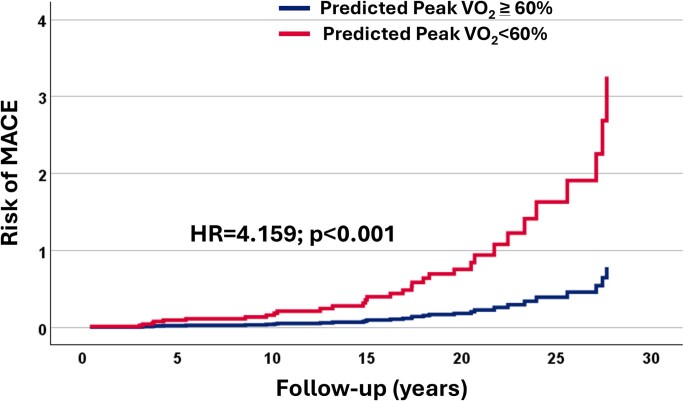
Risk of major adverse cardiac events based on peak VO_2_ in the overall population. MACE, major adverse cardiac events

After identifying the variables that were significantly different in the univariate analysis (*Table 3*), the multivariable Cox regression analysis revealed that NYHA Class >I and a *P*VO_2_% < 60 were significant independent predictors of MACE [adjusted hazard ratio (aHR) 2.273, 95% confidence interval (CI) 1.057–4.891, *P* = .036; aHR 4.159, 95% CI 1.894–9.137, *P* < .001, respectively] (*Table 3*, *Figure 1*, and *Graphical Abstract*). In a separate adjusted Cox model, we investigated the independent effect of continuous *P*VO_2_% and found that every 10% decrease in *P*VO_2_ corresponds to a 45% increased risk of combined MACE (aHR 1.449, 95% CI 1.136–1.848, *P* = .002) (*Table 3*). An additional separate adjusted model including the *P*VO_2_% spline term with knots at 20%, 40%, 60%, and 80% revealed that the non-linear term was not significant (Wald test *P* = .461).

**Table 3:** Predictors of major adverse cardiac events at univariate and multivariate analysis

Variable	Univariate analysis			Multivariate analysis		
	HR	95% CI	*P*	HR	95% CI	*P*
Age (years)	1.018	0.995–1.041	.119			
Age at diagnosis	1.010	0.989–1.030	.349			
Female sex	1.225	0.607–2.474	.571			
Family history of HCM	0.655	0.321–1.336	.245			
Family history of SD	1.074	0.560–2.061	.830			
Hypertension	1.118	0.435–2.877	.817			
Obesity	0.596	0.183–1.945	.391			
Sarcomeric mutation	1.374	0.507–3.720	.532			
NYHA functional Class >I	2.951	1.548–5.624	.001	2.273	1.057–4.891	.036
LAVi (≥34 ml/m^2^)	3.091	1.551–6.158	.001	1.667	0.754–3.685	.207
LV MWT (mm)	1.060	1.007–1.116	.027	1.015	0.953–1.081	.646
LV ejection fraction (%)	0.993	0.918–1.075	.869			
LVOTO (≥30 mmHg)	3.637	1.765–7.494	<.001	2.156	0.883–5.267	.092
Peak VO_2_ (ml/kg/min)	0.903	0.854–0.954	<.001			
Percentage predicted peak VO_2_ (%)^a^	1.443	1.170–1.752	<.001	1.449^b^	1.136–1.848^b^	.002^b^
Percentage predicted peak VO_2_ < 60%	4.329	2.144–8.744	<.001	4.159	1.894–9.137	<.001

HCM, hypertrophic cardiomyopathy; LAVi, indexed left atrial volume; LV, left ventricular; LVOTO, left ventricular outflow tract obstruction; MACE, major adverse cardiac events; MWT, maximal wall thickness; NYHA, New York Heart Association; Peak VO_2_, peak oxygen consumption; SD, sudden death.

^a^All HRs reported per 10% points increase.

^b^Estimated with a separate model adjusted by NYHA, LAVi, LV MWT, and LVOTO.

**Figure 2:**
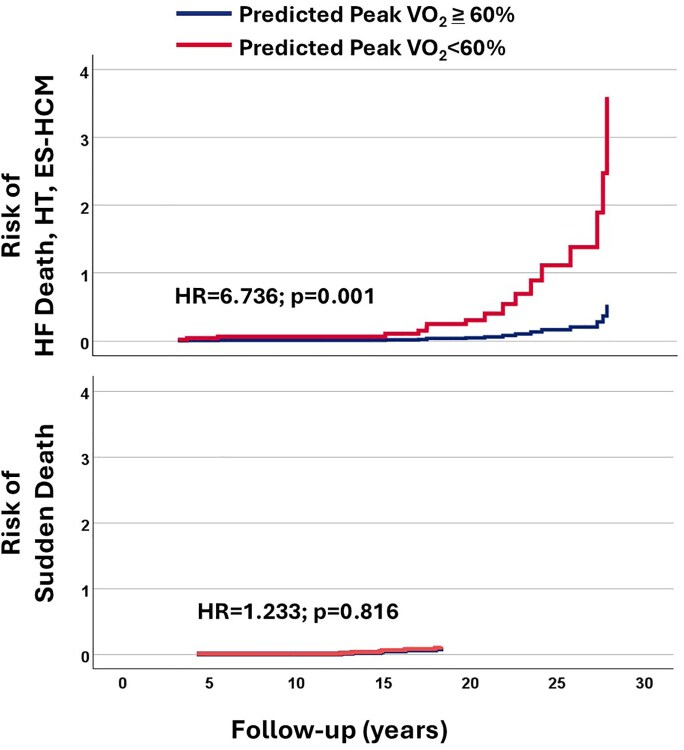
Adjusted hazard curves for heart failure–related events and sudden death according to *P*VO_2_% category. HF, heart failure; HT, heart transplantation; ES-HCM, end-stage hypertrophic cardiomyopathy

As secondary analysis, we disaggregated the composite endpoint into HF-related events (HFdeath/HT/ES-HCM) and SCD. We found that *P*VO_2_% < 60 was a strong predictor of HF-related events (HF death/HT/ES-HCM) [hazard ratio (HR) 6.736; 95% CI 2.130–21.304; *P* = .001], whereas it was no longer predictive for the SD outcome (HR 1.223; 95% CI 0.225–6.653; *P* = .816) (*Figure 2*, Supplementary Figure S1).

Adding in a separate adjusted model, the interaction between *P*VO_2_% < 60 and LVOTO revealed that such a term was not significant (interaction term *P* = .757). Among the 88 patients without LVOTO, both LAVi >34 ml/m^2^ and *P*VO_2_% < 60 were independently associated with MACE (aHR 5.25, 95% CI 1.09–25.37, *P* = .039 and aHR 6.06, 95% CI 1.24–29.73, *P* = .026, respectively). In contrast, among the 66 patients with LVOTO, *P*VO_2_% < 60 was the only independent predictor of MACE (aHR 3.47, 95% CI 1.38–8.69, *P* = .008).

The analysis was repeated, excluding patients who had had SRT. However, in two of them, SRT preceded the development of ES-HCM, whereas in an additional patient, it preceded death for HF. Thus, these three patients were included, as indicated in the statistical analysis, using SRT as a competing risk. Interestingly, in this analysis, the only predictor of MACE became *P*VO_2_% < 60 (aHR 3.966, 95% CI 1.626–9.670; *P* = .002).

**Figure 3:**
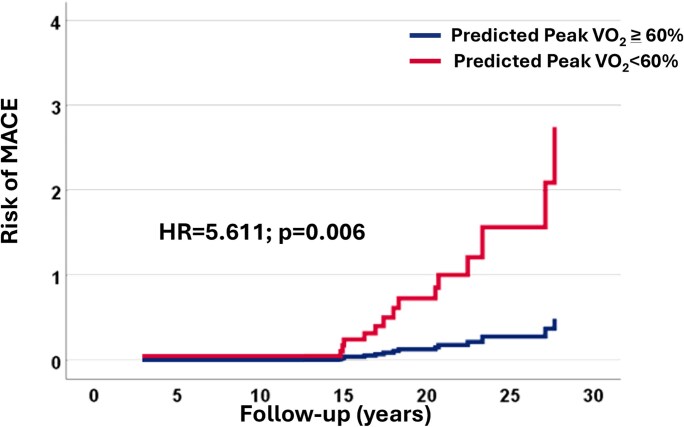
Risk of major adverse cardiac events based on peak VO_2_ in asymptomatic patients. MACE, major adverse cardiac events

We subsequently performed a multivariable analysis to assess whether the prognostic value of a *P*VO_2_% < 60 extended to the subgroup of 98 asymptomatic patients. Of these, 23 m (24%) showed *P*VO_2_% < 60. Eighteen patients developed MACE. In this subgroup, a *P*VO_2_% < 60 remained the only significant predictor of adverse outcomes. Specifically, it was associated with a five-fold increased risk of MACE (aHR 5.611, 95% CI 1.635–19.253, *P* = .006) (*Table 4*, *Figure 1*, left panel, and *Graphical Abstract*). Notably, in asymptomatic patients, risk differences emerged after ∼15 years of follow-up (*Figure 3* and *Graphical Abstract*). In this group, only one patient had SRT, which, however, preceded the development of ES-HCM. The competing risk analysis confirmed that a *P*VO_2_% < 60 was the only predictor of MACE.

**Table 4:** Multivariate analysis in asymptomatic patients at baseline

Variable	HR	95% CI	*P*
LAVi (≥34 ml/m^2^)	1.865	0.623–5.578	.265
Maximal LV wall thickness (mm)	1.070	0.969–1.181	.182
LV outflow tract obstruction (≥30 mmHg)	1.530	0.502–4.662	.455
Percentage predicted peak VO_2_ < 60%	5.611	1.635–19.253	.006

LAVi, indexed left atrial volume.

## Discussion

This study underscores the critical prognostic value of *P*VO_2_% in HCM, representing the first to do so over such an extended follow-up period. A moderate to severe reduction in exercise capacity, defined as a *P*VO_2_% < 60, was independently associated with an increased risk of MACE, along with more advanced symptomatic status (NYHA Class ≥II). Importantly, among patients classified as NYHA Class I, a *P*VO_2_% < 60 emerged as the sole predictor of MACE.

Cardiopulmonary exercise testing is a well-established, non-invasive method to evaluate the integrated performance of the cardiovascular and pulmonary systems during exertion. In HCM, exercise capacity is impaired by diastolic dysfunction, myocardial ischaemia, and LVOTO, all of which stem from characteristic histopathologic features such as myocyte hypertrophy, disarray, hypercontractility, interstitial fibrosis, and microvascular dysfunction.^5,6,10,25^ These abnormalities reduce exercise tolerance by limiting stroke volume augmentation, disrupting ventilation-perfusion matching, and impairing peripheral oxygen utilization.^20,21,25^ Although peak VO_2_ has demonstrated prognostic relevance, most previous studies were limited by analysing patients with obstructive HCM.^6,9,10,23,26–28^ In patients with obstructive HCM and over a mean follow-up of 4.0 years, Sorajja *et al*. demonstrated that reduced exercise capacity, defined as a *P*VO_2_% < 60 and an absolute VO_2_ < 18 ml/kg/min, was independently associated with an increased risk of death or progression to severe cardiac symptoms. Again, in a cohort of obstructive HCM patients undergoing septal myectomy, *P*VO_2_% < 60 was also identified as a predictor of increased overall mortality.^23^ In contrast, our study evaluated a broader HCM population, including both obstructive and nonobstructive forms, as well as symptomatic and asymptomatic individuals, additionally, with a notably longer follow-up period. In our cohort, reduced *P*VO_2_% was an independent determinant of outcome in both obstructive and nonobstructive patients. In nonobstructive HCM, diastolic dysfunction appears to be the predominant pathophysiological mechanism, which may explain why LAVi, reflecting impaired diastolic filling, was also independently associated with adverse outcomes. In contrast, in obstructive patients, LVOTO is the main pathophysiological driver, and *P*VO_2_%, reflecting limited cardiac output during exercise, remained the primary predictor of outcome.

Another novelty of our findings lies in the definition of the endpoint, which included only MACE, deliberately excluding non-cardiac deaths. In a large cohort of 1898 HCM patients followed for a mean of 5.6 years, Coats *et al*.^9^ demonstrated that peak VO_2_ was an independent predictor of overall mortality and cardiac transplantation. However, their endpoint included non-cardiac deaths, which may be influenced by comorbidities unrelated to HCM.^9^ In contrast, our study aimed to isolate the specific prognostic value of peak VO_2_ in predicting HCM-related outcomes, thus providing a more disease-focused risk assessment.

Of note, we included SRT in the MACE definition, an intervention that, while technically a treatment, reflects progression to advanced disease. To account for this and better clarify the prognostic role of peak VO_2_, we performed a competing risk analysis treating SRT as a competing event, confirming the prognostic role of VO_2_. This allowed us to distinguish the risk of adverse outcomes from the decision to undergo invasive therapy.

In addition, our secondary analysis suggests that CPET is more predictive of HF development than of SD. This is likely because CPET reflects the cumulative haemodynamic burden on the LV, resulting from obstruction, mitral regurgitation, ischaemia, and diastolic dysfunction, which may ultimately lead to HF progression, rather than the arrhythmic substrate underlying SD. These results are consistent with previous evidence on the prognostic performance of CPET in broader cohorts of patients with HCM.^9^

Our findings gain further relevance when considered in the context of current clinical recommendations. The latest European Society of Cardiology (ESC) guidelines on HCM align with the American College of Cardiology/American Heart Association (ACC/AHA) recommendations, strongly recommend conducting CPET only in symptomatic and severely symptomatic HCM patients to consider advanced HF therapies, such as HT. For asymptomatic patients, the guidelines suggest CPET with a Class IIb recommendation.^2,11,12^ However, CPET, especially when combined with echocardiography, should be considered a fundamental component of the initial evaluation in all HCM patients, irrespective of symptom status. Numerous studies have shown that a reduced peak VO_2_ is common across HCM without overt symptoms. Approximately 70% of patients classified as NYHA Class I have been found to exhibit abnormal peak VO_2_ values.^20^ This finding is further supported by the VANISH trial, which enrolled patients with early-stage and subclinical sarcomeric HCM, the majority of whom were classified as NYHA Class I. Despite their young age and seemingly stable clinical profile, a significant proportion of these individuals demonstrated reduced peak VO_2_, revealing underlying functional impairment not apparent through symptoms alone.^29,30^ Thus, it is not surprising that in our cohort, almost one in four asymptomatic patients showed a *P*VO_2_% < 60.

In addition, we confirm that patients in NYHA Class I should not necessarily be considered to have a favourable prognosis. Recently, Ahluwalia *et al*.^31^ reported that 28% of obstructive HCM patients classified as NYHA Class I experienced adverse events over 5 years, with age, female sex, and left atrial enlargement emerging as independent predictors. Thus, we further suggest the need to perform CPET even in asymptomatic patients to better stratify risk in HCM.

### Study limitations

This study was observational, conducted at a single centre, and utilized a retrospective design with a relatively small sample size. However, it benefits from a long follow-up period. We included only patients who were physically capable of exercising, thereby excluding individuals classified as NYHA Class IV. Additionally, patients with an LVEF below 50% were not included. In our analysis, the VE/VCO_2_ slope did not show a significant difference between patients who experienced MACE and those who did not. This finding may be explained by the relatively low proportion of severely symptomatic patients in our cohort, likely influenced by the exclusion of individuals with reduced LVEF and chronic coronary syndrome. Because of the limited number of patients who underwent CMR, we could not evaluate the independent contribution of VO_2_ and LGE to clinical outcomes. Notably, this specific question has not yet been explored in existing literature.

## Conclusions

The role of CPET in the prognostic assessment of patients with HCM is not yet fully established. Our study, with a long-term follow-up, helps fill this gap by showing that CPET provides valuable prognostic information not only in symptomatic patients but also, importantly, in those who are asymptomatic. Cardiopulmonary exercise testing can identify a subset of NYHA Class I patients who, despite appearing clinically stable, have an increased long-term risk of adverse events. These findings highlight the potential value of CPET as part of a multimodal assessment strategy in HCM, enabling early identification of high-risk individuals who may benefit from closer monitoring and timely intervention.

## Supplementary Material

xvag013_Supplementary_Data

## Declarations

### Data Availability

No data were generated or analysed for this manuscript.

### Pre-registered Clinical Trial Number

None supplied.

## References

[1] Marian AJ, Braunwald E. Hypertrophic cardiomyopathy: genetics, pathogenesis, clinical manifestations, diagnosis, and therapy. Circ Res 2017;121:749–70. 10.1161/CIRCRESAHA.117.311059 28912181 PMC5654557

[2] Arbelo E, Protonotarios A, Gimeno JR, Arbustini E, Barriales-Villa R, Basso C, et al 2023 ESC guidelines for the management of cardiomyopathies: developed by the task force on the management of cardiomyopathies of the European Society of Cardiology (ESC). Eur Heart J 2023;44:3503–626. 10.1093/eurheartj/ehad194 37622657

[3] Elliott PM, Gimeno JR, Thaman R, Shah J, Ward D, Dickie S, et al Historical trends in reported survival rates in patients with hypertrophic cardiomyopathy. Heart 2006;92:785–91. 10.1136/hrt.2005.068577 16216855 PMC1860645

[4] Jung H, Sung JH, Yang PS, Jang E, Yu HT, Kim TH, et al Stroke risk stratification for atrial fibrillation patients with hypertrophic cardiomyopathy. J Am Coll Cardiol 2018;72:2409–11. 10.1016/j.jacc.2018.07.098 30384898

[5] Balady GJ, Arena R, Sietsema K, Myers J, Coke L, Fletcher GF, et al Clinician’s guide to cardiopulmonary exercise testing in adults. Circulation 2010;122:191–225. 10.1161/CIR.0b013e3181e52e69 20585013

[6] Writing Committee, EACPR; Guazzi M, Adams V, Conraads V, Halle M, et al Clinical recommendations for cardiopulmonary exercise testing data assessment in specific patient populations. Eur Heart J 2012;33:2917–27. 10.1093/eurheartj/ehs221 22952138

[7] Sharma S, Elliott PM, Whyte G, Mahon N, Virdee MS, Mist B, et al Utility of metabolic exercise testing in distinguishing hypertrophic cardiomyopathy from physiologic left ventricular hypertrophy in athletes. J Am Coll Cardiol 2000;36:864–70. 10.1016/S0735-1097(00)00816-0 10987612

[8] Ciampi Q, Betocchi S, Losi MA, Ferro A, Cuocolo A, Lombardi R, et al Abnormal blood-pressure response to exercise and oxygen consumption in patients with hypertrophic cardiomyopathy. J Nucl Cardiol 2007;14:869–75. 10.1016/j.nuclcard.2007.08.003 18022114

[9] Coats CJ, Rantell K, Bartnik A, Patel A, Mist B, McKenna WJ, et al Cardiopulmonary exercise testing and prognosis in hypertrophic cardiomyopathy. Circ Heart Fail 2015;8:1022–31. 10.1161/CIRCHEARTFAILURE.114.002248 26374874

[10] Rowin EJ, Maron BJ, Olivotto I, Maron MS. Role of exercise testing in hypertrophic cardiomyopathy. JACC Cardiovasc Imaging 2017;10:1374–86. 10.1016/j.jcmg.2017.07.016 29122139

[11] Ommen SR, Ho CY, Asif IM, Balaji S, Burke MA, Day SM, et al 2024 AHA/ACC/AMSSM/HRS/PACES/SCMR guideline for the management of hypertrophic cardiomyopathy. J Am Coll Cardiol 2024;83:2324–405. 10.1016/j.jacc.2024.02.014 38727647

[12] Authors/Task Force Members; Elliott PM, Anastasakis A, Borger MA, Borggrefe M, Cecchi F, et al 2014 ESC guidelines on diagnosis and management of hypertrophic cardiomyopathy: the task force for the diagnosis and management of hypertrophic cardiomyopathy of the European Society of Cardiology (ESC). Eur Heart J 2014;35:2733–79. 10.1093/eurheartj/ehu284 25173338

[13] Nagueh SF, Phelan D, Abraham T, Armour A, Desai MY, Dragulescu A, et al Recommendations for multimodality cardiovascular imaging of patients with hypertrophic cardiomyopathy: an update from the American Society of Echocardiography, in Collaboration with the American Society of Nuclear Cardiology, the Society for Cardiovascular Magnetic Resonance, and the Society of Cardiovascular Computed Tomography. J Am Soc Echocardiogr 2022;35:533–69. 10.1016/j.echo.2022.03.012 35659037

[14] Landrum MJ, Lee JM, Benson M, Brown GR, Chao C, Chitipiralla S, et al ClinVar: improving access to variant interpretations and supporting evidence. Nucleic Acids Res 2018;46:D1062–7. 10.1093/nar/gkx1153 29165669 PMC5753237

[15] Losi MA, Imbriaco M, Canciello G, Pacelli F, Di Nardo C, Lombardi R, et al Left ventricular mass in hypertrophic cardiomyopathy assessed by 2D-echocardiography: validation with magnetic resonance imaging. J Cardiovasc Transl Res 2020;13:238–44. 10.1007/s12265-019-09911-3 31489577

[16] Borrelli F, Lombardi R, Canciello G, Frisso G, Todde G, Esposito G, et al Mechano-energetic efficiency in patients with hypertrophic cardiomyopathy with and without sarcomeric mutations. J Cardiovasc Transl Res 2024;17:458–66. 10.1007/s12265-023-10441-2 37833437

[17] Briguori C, Betocchi S, Romano M, Manganelli F, Angela Losi M, Ciampi Q, et al Exercise capacity in hypertrophic cardiomyopathy depends on left ventricular diastolic function. Am J Cardiol 1999;84:309–15. 10.1016/S0002-9149(99)00282-9 10496441

[18] Maron BJ, Desai MY, Nishimura RA, Spirito P, Rakowski H, Towbin JA, et al Management of hypertrophic cardiomyopathy: JACC state-of-the-art review. J Am Coll Cardiol 2022;79:390–414. 10.1016/j.jacc.2021.11.021 35086661

[19] Al-Khatib SM, Stevenson WG, Ackerman MJ, Bryant WJ, Callans DJ, Curtis AB, et al 2017 AHA/ACC/HRS guideline for management of patients with ventricular arrhythmias and the prevention of sudden cardiac death: executive summary: a report of the American College of Cardiology/American Heart Association Task Force on Clinical Practice Guidelines and the Heart Rhythm Society. Heart Rhythm 2018;15:e190–252. 10.1016/j.hrthm.2017.10.035 29097320

[20] Sharma S, Elliott P, Whyte G, Jones S, Mahon N, Whipp B, et al Utility of cardiopulmonary exercise in the assessment of clinical determinants of functional capacity in hypertrophic cardiomyopathy. Am J Cardiol 2000;86:162–8. 10.1016/S0002-9149(00)00854-7 10913477

[21] Robbins M, Francis G, Pashkow FJ, Snader CE, Hoercher K, Young JB, et al Ventilatory and heart rate responses to exercise. Circulation 1999;100:2411–7. 10.1161/01.CIR.100.24.2411 10595953

[22] Sorajja P, Allison T, Hayes C, Nishimura RA, Lam CSP, Ommen SR. Prognostic utility of metabolic exercise testing in minimally symptomatic patients with obstructive hypertrophic cardiomyopathy. Am J Cardiol 2012;109:1494–8. 10.1016/j.amjcard.2012.01.363 22356797

[23] Cui H, Schaff HV, Olson TP, Geske JB, Dearani JA, Nishimura RA, et al Cardiopulmonary exercise test in patients with obstructive hypertrophic cardiomyopathy. J Thorac Cardiovasc Surg 2024;167:701–10.e3. 10.1016/j.jtcvs.2022.05.025 35798610

[24] Losi MA, Izzo R, Mancusi C, Wang W, Roman MJ, Lee ET, et al Depressed myocardial energetic efficiency increases risk of incident heart failure: the strong heart study. J Clin Med 2019;8:1044. 10.3390/jcm8071044 31319598 PMC6678469

[25] Magrì D, Santolamazza C. Cardiopulmonary exercise test in hypertrophic cardiomyopathy. Ann Am Thorac Soc 2017;14:S102–9. 10.1513/AnnalsATS.201611-884FR 28375659

[26] Masri A, Pierson LM, Smedira NG, Agarwal S, Lytle BW, Naji P, et al Predictors of long-term outcomes in patients with hypertrophic cardiomyopathy undergoing cardiopulmonary stress testing and echocardiography. Am Heart J 2015;169:684–92.e1. 10.1016/j.ahj.2015.02.006 25965716

[27] Magrì D, Re F, Limongelli G, Agostoni P, Zachara E, Correale M, et al Heart failure progression in hypertrophic cardiomyopathy– possible insights from cardiopulmonary exercise testing. Circ J 2016;80:2204–11. 10.1253/circj.CJ-16-0432 27628102

[28] Finocchiaro G, Haddad F, Knowles JW, Caleshu C, Pavlovic A, Homburger J, et al Cardiopulmonary responses and prognosis in hypertrophic cardiomyopathy: a potential role for comprehensive noninvasive hemodynamic assessment. JACC Heart Fail 2015;3:408–18. 10.1016/j.jchf.2014.11.011 25863972

[29] Vissing CR, Axelsson Raja A, Day SM, Russell MW, Zahka K, Lever HM, et al Cardiac remodeling in subclinical hypertrophic cardiomyopathy: the VANISH randomized clinical trial. JAMA Cardiol 2023;8:1083–8. 10.1001/jamacardio.2023.2808 37672268 PMC10483382

[30] Ireland CG, Burstein DS, Day SM, Axelsson Raja A, Russell MW, Zahka KG, et al Quality of life and exercise capacity in early stage and subclinical hypertrophic cardiomyopathy: a secondary analysis of the VANISH trial. Circ Heart Fail 2024;17:e011663. 10.1161/CIRCHEARTFAILURE.124.011663 39087355 PMC11335449

[31] Ahluwalia M, Liu J, Olivotto I, Parikh V, Ashley EA, Michels M, et al The clinical trajectory of NYHA functional class I patients with obstructive hypertrophic cardiomyopathy. JACC Heart Fail 2025;13:332–43. 10.1016/j.jchf.2024.09.008 39520446

