# Supplementary figures and images for: Long-term prognostic value of cardiopulmonary exercise testing in patients with hypertrophic cardiomyopathy
Source: ESC Heart Fail. 2026 Jan 22;13(1):xvag013. doi: 10.1093/eschf/xvag013 (PMC13108259; doi:10.1093/eschf/xvag013)

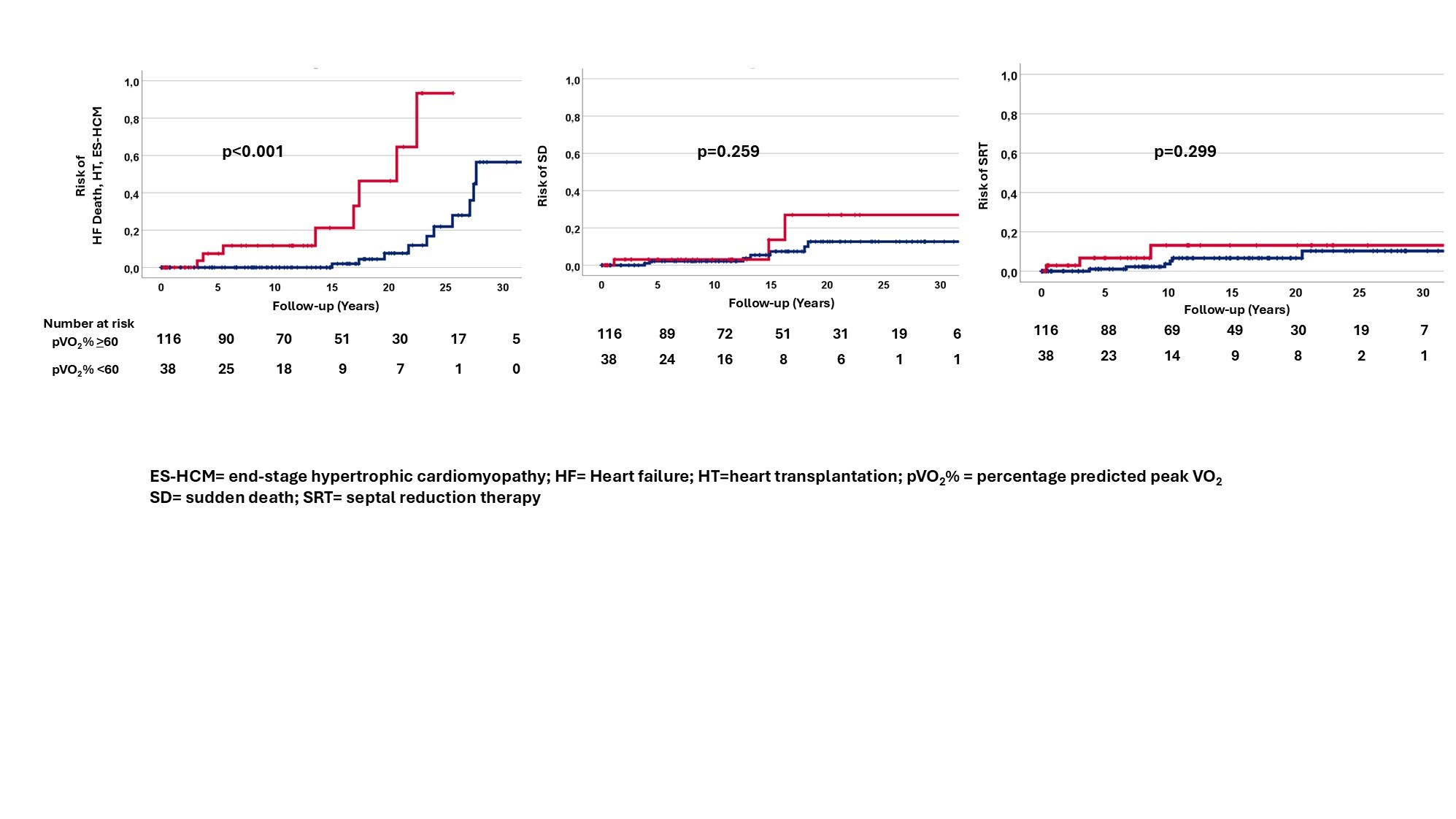

Supplement: xvag013_Supplementary_Data [file xvag013_supplementary_data.jpeg]
